# Weaker Response to XueBiJing Treatment in Severe Community-Acquired Pneumonia Patients With Higher Body Mass Index or Hyperglycemia: A *Post Hoc* Analysis of a Randomized Controlled Trial

**DOI:** 10.3389/fphar.2022.755536

**Published:** 2022-06-03

**Authors:** Yansha Song, Xiaocen Wang, Cuicui Chen, Tingting Wei, Ke Lang, Dong Yang, Yuanlin Song

**Affiliations:** Department of Pulmonary and Critical Care Medicine, Zhongshan Hospital, Fudan University, Shanghai, China

**Keywords:** severe community-acquired pneumonia, post hoc analysis, randomized controlled trial, 28-day mortality rate, XueBiJing injection

## Abstract

**Background:** Overweight and hyperglycemia might result in poor prognosis in patients with severe community-acquired pneumonia (SCAP). XueBiJing treatment could significantly improve the outcomes of patients with SCAP. We investigated the efficacy of XueBiJing injection in patients with SCAP stratified by body mass index (BMI) and fasting blood glucose (FBG).

**Methods:** This is a *post hoc* analysis of XueBiJing trial, a large prospective, randomized, controlled study conducted in 33 hospitals in China. We compared data from non-overweight (BMI <24 kg/m^2^, *n* = 425) vs. overweight (BMI ≥24 kg/m^2^, *n* = 250) patients as well as non-hyperglycemia (FBG <7 mmol/L, *n* = 315) vs. hyperglycemia (FBG ≥7 mmol/L, *n* = 360) patients with XueBiJing, 100 ml, q12 h, or a visually indistinguishable placebo treatment for 5–7 days.

**Results:** Among patients with BMI <24 kg/m^2^ (*n* = 425), 33 (15.3%), XueBiJing recipients and 52 (24.9%) placebo recipients (*p* = 0.0186) died within 28 days. Among patients with BMI ≥24 kg/m^2^ (*n* = 250), XueBiJing recipients still had lower mortality (XueBiJing 16.9% vs. placebo 24.2%; *p* = 0.2068) but without significantly statistical difference. For the FBG group, patients with FBG <7 mmol/L (*n* = 315), 18 (11.2%) XueBiJing recipients and 32 (20.8%) placebo recipients (*p* = 0.030) died within 28 days. Among patients with FBG ≥7 mmol/L (*n* = 360), XueBiJing recipients still had lower mortality (XueBiJing 20.2% vs. placebo 27.8%; *p* = 0.120) but without significantly statistical difference. The total duration of the ICU stay and the duration of mechanical ventilation were similar in both groups (*p* > 0.05).

**Conclusion:** Overweight or hyperglycemia might weaken the efficacy of XueBiJing injection in the treatment of SCAP as indicated by the significant elevated risk of 28-day mortality. Additional studies are needed to validate our findings and to further understand the underlying mechanisms.

## Background

Community-acquired pneumonia remains one of the leading infectious diseases despite advances in antibiotic treatment (Torres et al., 2015). The mortality rate among patients admitted to hospital with pneumonia is estimated at 8–15% (Fine et al., 1996). The main physiological function of the lung is gas exchange. At the same time, because it is directly exposed to the outside world, the lung is vulnerable to microbial invasion and environmental noxious particles (Hu et al., 2021). In healthy lungs, this defense function is mainly achieved through the production of epithelial barriers and surfactant proteins by alveolar cells (Ruaro et al., 2021). Obesity and hyperglycemia were reported as risk factors for poor prognosis of acquiring infections including community-acquired pneumonia (CAP). Obesity may influence either the risk of getting an infection or the outcome of an infection once it is established. Obese people are more likely than people of normal weight to develop serious complications of respiratory infections (Falagas and Kompoti, 2006; Kornum et al., 2010; Huttunen and Syrjänen, 2013). A recent large observational study suggested a stepwise increase in mortality and length of hospital stay in CAP patients with higher serum glucose levels (Lepper et al., 2012). Hyperglycemia was demonstrated to be a common occurrence during sepsis and independently associated with increased mortality in sepsis patients (van Vught et al., 2016). Clinical trials in patients with CAP in different nutritional and metabolic status are lacking. But, it is unclear whether obesity and hyperglycemia impact the choices of treatments and the effectiveness of drugs in CAP patients, not to mention patients with severe CAP (SCAP).

XueBiJing, an injectable prescription from traditional Chinese medicine, has been approved to treat severe infections (sepsis) in guidelines (China Food and Drug Administration, Beijing, China, Number Z20040033) (Qi et al., 2011; Li et al., 2020). Animal experiments and clinical research studies showed that XueBiJing combined with conventional therapy could improve the prognosis of patients with infectious diseases (Chen et al., 2018; Li et al., 2018; Li et al., 2021). More inspiringly, it was recently reported that XueBiJing injection led to a statistically significant improvement in the mortality, duration of mechanical ventilation, and duration of ICU stay in critically ill patients with SCAP (Song et al., 2019).

In consideration of the impact of overweight and hyperglycemia on the poor prognosis of CAP and the effects of XueBiJing on SCAP patients, the aim of this study was to investigate the impact of obesity and hyperglycemia on outcomes in patients with SCAP by *post hoc* analysis of the large, randomized, controlled XueBiJing trial in order to provide related data for physicians to make optimal clinical decision. We recommend XueBiJing for non-overweight and non-hyperglycemic patients due to the significant reduction in 28-day mortality.

## Materials and Methods

### Participants and Study Design

This study is a *post hoc* analysis of the main multicenter randomized controlled trial (RCT), “XueBiJing injection versus placebo for critically ill patients with severe community-acquired pneumonia: a randomized controlled trial.” The study design and results have been published in detail previously (Song et al., 2019). The study was approved by the Medical Ethics Committee of Zhongshan Hospital, Fudan University [2011–2038 (Hu et al., 2021)], and informed consents were obtained from all patients or their legal guardians before enrollment. Eligible 710 participants enrolled at 33 centers with SCAP were randomly administered with XueBiJing (*n* = 334) or placebo (*n* = 341) for 5–7 days. The participants were followed up for 28 days after randomization. The participants received the solvent only (normal saline, 200 ml, q12 h) in the placebo group and the solvent plus XueBiJing (normal saline 100 ml + XueBiJing 100 ml, q12 h) in the XueBiJing group. Both groups received a standard therapy (such as antibiotics) chosen by the attending physician according to the 2007 American Thoracic Society/Infectious Diseases Society of America guidelines (Mandell et al., 2007). Patients were categorized into two different BMI groups or FBG groups: non-overweight (BMI <24.0 kg/m^2^) and overweight (BMI ≥24.0 kg/m^2^) based on the Criteria of Weight for Adults released by the Ministry of Health of China and previous study (Liu et al., 2018; Dong et al., 2019; Wang et al., 2019) or non-hyperglycemia (FBG ≥7 mmol/L) and hyperglycemia (FBG ≥7 mmol/L) according to the international standard (Menke et al., 2015; American Diabetes Association, 2021).

### Endpoints

In the XueBiJing study, an 8-day improvement in pneumonia severity index (PSI) risk rating was defined as the primary outcome. Secondary outcomes included 28-day mortality, the duration of mechanical ventilation, and the total duration of ICU stay. In this *post hoc* analysis, the primary outcome was 28-day mortality. The main secondary outcomes were the duration of mechanical ventilation and total duration of ICU stay.

### Statistical Analysis

Continuous variables were expressed as mean ± standard deviation (SD) or median with interquartile range (IQR), while categorical variables were expressed as frequencies with percentages. Primary outcome analysis was a simple categorical frequency comparison by use of the chi-squared test. The secondary outcomes for the duration of mechanical ventilation and total duration of ICU stay were analyzed by the *t*-test or the Wilcoxon rank sum test as appropriate. Kaplan–Meier estimates were used for time-to-event variables, and differences among the groups were compared with a log rank test. HR and associated 95% CIs were estimated from the Cox proportional hazard model. All the analyses were performed on the modified intention-to-treat population, which comprised all the patients who were randomized to treatment. A two-tailed value of *p* < 0.05 was regarded as significant. All analyses were performed with R version 4.0.3.

## Results

**FIGURE 1:**
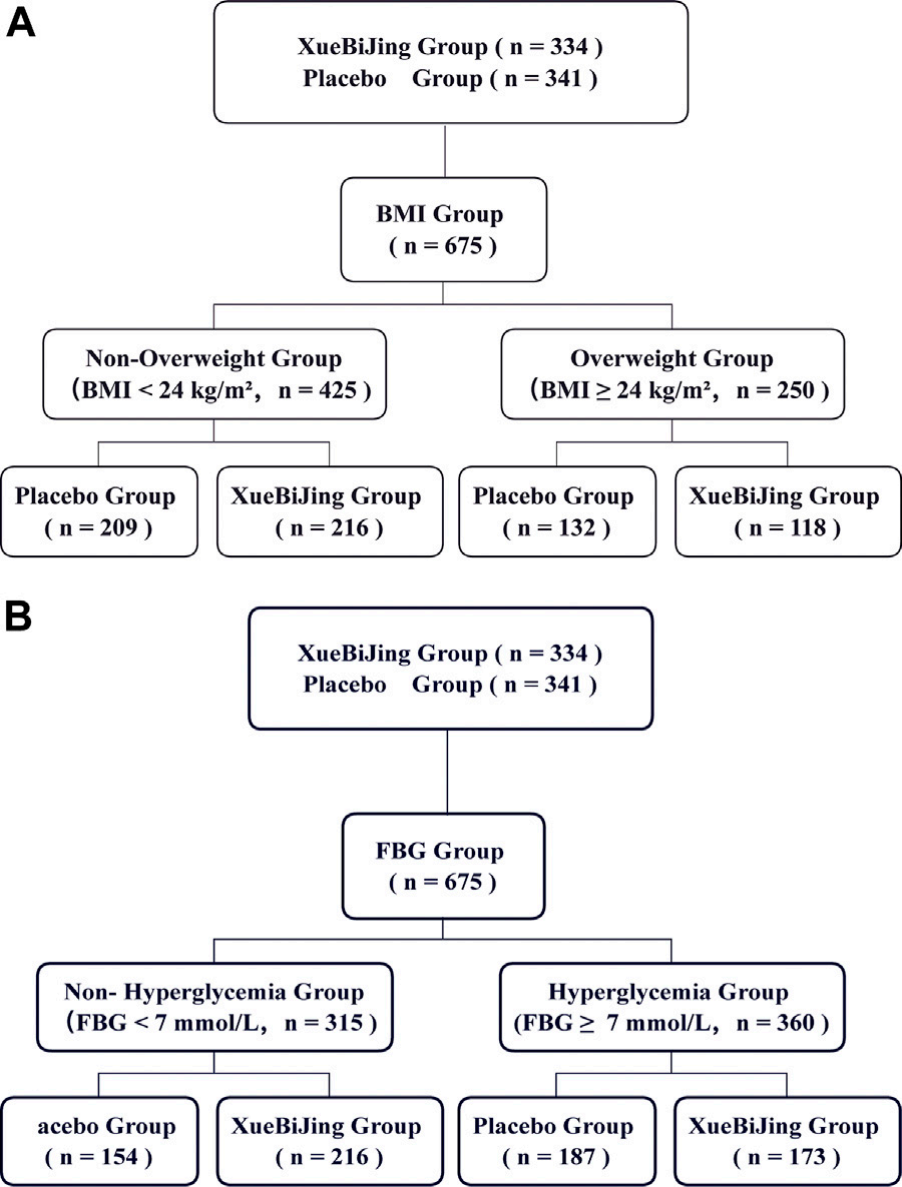
The profile of our Study: **(A)** BMI Group; **(B)** FBG Group.

Of the 710 participants enrolled at 33 centers, 334 were assigned to the XueBiJing treatment group and 341 to the placebo treatment group; 35 participants were excluded from this study due to missing data. Figure 1 shows the study profile of our study. Demographic and basal clinical characteristics of all participants are listed in Tables 1, 2, which was classified according to BMI or FBG. The BMI of overweight patients (*n* = 250) was 26.28 ± 2.15 kg/m^2^, and 68.8% of them (*n* = 172) were male. The FBG of hyperglycemia patients (*n* = 360) was 10.63 ± 3.67 mmol/L, and 71.7% of them (*n* = 258) were male. Differences of baseline comorbidities, PSI score, or other biological parameters were not significantly different between high and not-high BMI or high and not-high FBG groups. In Tables 3, 4, the proportion of patients with ARDS and the baseline settings of mechanical ventilation was not significantly different from that of the compared group either.

**TABLE 1:** Demographic and basal clinical characteristics of patients between XueBiJing and placebo groups in the BMI group.

	Non-overweight (BMI <24)		*p*-value	Overweight (BMI ≥24)		*p*-value
	Placebo group	XBJ group		Placebo group	XBJ group	
N	209	216		132	118	
Age, yr,*	58.18 (15.52)	59.12 (13.82)	0.508	58.05 (11.98)	57.85 (13.15)	0.897
Men, %	148 ( 70.8)	138 ( 63.9)	0.156	86 ( 65.2)	86 ( 72.9)	0.238
Body mass index, kg/m^2^,*	20.73 (2.95)	20.71 (2.84)	0.943	26.15 (2.13)	26.42 (2.16)	0.333
Systolic blood pressure, mm Hg, *	128.81 (24.96)	127.35 (21.45)	0.519	130.85 (20.89)	133.96 (25.61)	0.292
Heart rate, beats/min, *	108.34 (20.11)	109.63 (20.50)	0.513	108.28 (18.89)	110.98 (20.99)	0.285
Respiratory rate, breaths/min, *	27.89 (6.63)	27.77 (6.82)	0.852	27.92 (5.44)	28.47 (5.22)	0.424
Temperature, °C, *	37.97 (1.06)	38.02 (1.05)	0.624	38.17 (1.04)	38.28 (1.08)	0.419
Pao2/Fio2, *	173.88 (55.85)	170.94 (56.81)	0.592	171.97 (53.73)	164.47 (54.47)	0.275
Glasgow score,*	11.53 (3.53)	11.40 (3.55)	0.709	11.89 (3.58)	12.14 (3.17)	0.574
Comorbidities (%)						
Chronic bronchitis	4 (1.9)	1 (0.46)		0 ( 0.0)	3 (2.54)	
Coronary artery disease	3 ( 1.4)	4 (1.85)		5 (3.79)	3 (2.54)	
Diabetes mellitus (any type)	5 ( 2.39)	5 (2.31)		3 (2.27)	6 (5.08)	
Hypertension	30 (14.35)	18 (8.33)		18 (13.6)	20 (16.9)	
Parkinson’s disease	1 ( 0.48)	1 (0.46)		2 (1.51)	0 ( 0.0)	
Polytrauma	2 (0.96)	1 (0.46)		0 ( 0.0)	1 (0.85)	
C-reactive protein, mg/L, *	85.11 (75.32)	86.88 (84.71)	0.837	93.08 (110.15)	84.55 (110.17)	0.578
Leucocytes, 10^9^ cells/L, *	12.65 (6.93)	13.59 (6.98)	0.162	12.56 (5.86)	12.80 (6.62)	0.766
FBG, mmol/L, *	8.03 (3.42)	8.27 (3.93)	0.494	8.34 (3.90)	8.58 (3.74)	0.629
PT, *	14.91 (7.71)	14.47 (4.86)	0.493	14.14 (4.58)	13.71 (2.68)	0.389
PSI (%)						
Class I	7 ( 3.3)	7 ( 3.2)	0.846	7 ( 5.3)	3 ( 2.5)	0.511
Class II	10 ( 4.8)	13 ( 6.0)		10 ( 7.6)	9 ( 7.6)	
Class III	38 (18.2)	32 (14.8)		18 (13.6)	15 (12.7)	
Class IV	103 (49.3)	105 (48.6)		65 (49.2)	52 (44.1)	
Class V	51 (24.4)	59 (27.3)		32 (24.2)	39 (33.1)	
Total PSI score, *	114.74 (31.24)	116.42 (33.70)	0.602	112.34 (28.42)	115.63 (32.21)	0.401
APCHE II score, *	15.84 (6.61)	15.84 (6.17)	0.993	15.23 (5.96)	14.87 (6.10)	0.643
Mechanical ventilation, *n* (%)	129 (61.7)	130 (60.2)	0.822	84 (63.6)	80 (67.8)	0.577
SOFA score, *	6.29 (2.76)	6.16 (2.86)	0.631	5.77 (2.74)	5.97 (2.70)	0.561

PSI, pneumonia severity index. PSI risk class I corresponds to age ≤ 50 yr and no risk factors (≤ 50 points), risk class II to < 70 points, risk class III to 71–90 points, risk class IV to 91–130 points, and risk class V to > 130 points.

*mean (SD).

**TABLE 2:** Demographic and basal clinical characteristics of patients between XueBiJing and placebo groups in the FBG group.

	Non-hyperglycemia (FBG <7)		*p*-value	Hyperglycemia (FBG ≥7)		*p*-value
	Placebo group	XBJ group		Placebo group	XBJ group	
N	154	161		187	173	
Age, yr,*	56.27 (14.48)	57.63 (14.15)	0.399	59.66 (13.89)	59.64 (13.00)	0.988
Men, (%)	98 (63.6)	102 (63.4)	1	136 (72.7)	122 (70.5)	0.728
Body mass index, kg/m^2^, *	22.95 (3.86)	22.47 (3.65)	0.257	22.73 (3.67)	22.97 (3.89)	0.555
Systolic blood pressure, mm Hg, *	128.43 (21.36)	125.17 (19.59)	0.159	130.56 (25.07)	133.88 (25.44)	0.213
Heart rate, beats/min, *	108.05 (19.93)	107.59 (21.42)	0.845	108.55 (19.41)	112.46 (19.68)	0.059
Respiratory rate, breaths/min, *	27.84 (6.05)	28.73 (6.43)	0.204	27.96 (6.32)	27.35 (6.13)	0.354
Temperature, °C, *	38.15 (1.07)	38.27 (1.08)	0.302	37.96 (1.04)	37.96 (1.03)	0.969
Pao2/Fio2, *	169.83 (47.73)	169.62 (49.21)	0.969	175.86 (60.27)	167.75 (61.79)	0.208
Glasgow score, *	11.75 (3.40)	11.80 (3.34)	0.912	11.60 (3.67)	11.53 (3.53)	0.86
Comorbidities (%)						
Chronic bronchitis	2 (1.30)	1 (0.62)		2 (1.07)	3 (1.73)	
Coronary artery disease	4 (2.60)	3 (1.86)		4 (2.14)	4 (2.31)	
Diabetes mellitus (any type)	1 (0.65)	0 ( 0.0)		7 (3.74)	11 (6.36)	
Hypertension	14 (9.10)	16 (9.93)		34 (18.18)	22 (12.71)	
Parkinson’s disease	2 (1.30)	1 (0.62)		1 (0.53)	0 ( 0.0)	
Polytrauma	1 (0.65)	0 ( 0.0)		1 (0.53)	2 (1.16)	
C-reactive protein, mg/L, *	68.39 (91.49)	85.39 (107.19)	0.167	104.65 (85.24)	86.65 (80.53)	0.068
Leucocytes, 10^9^ cells/L, *	12.36 (5.95)	12.85 (6.29)	0.478	12.82 (6.98)	13.74 (7.34)	0.225
FBG, mmol/L, *	5.53 (0.91)	5.58 (0.87)	0.586	10.31 (3.58)	10.98 (3.74)	0.081
PT, *	13.90 (3.69)	13.99 (3.75)	0.835	15.21 (8.38)	14.39 (4.62)	0.26
PSI (%)						
Class I	8 ( 5.2)	6 ( 3.7)	0.952	6 ( 3.2)	4 ( 2.3)	0.242
Class II	13 ( 8.4)	13 ( 8.1)		7 ( 3.7)	9 ( 5.2)	
Class III	27 (17.5)	26 (16.1)		29 (15.5)	21 (12.1)	
Class IV	72 (46.8)	81 (50.3)		96 (51.3)	76 (43.9)	
Class V	34 (22.1)	35 (21.7)		49 (26.2)	63 (36.4)	
Total PSI score, *	107.92 (27.77)	110.58 (30.37)	0.43	118.59 (31.25)	121.24 (34.80)	0.453
APCHE II score, *	15.05 (6.52)	15.04 (5.82)	0.998	16.06 (6.22)	15.92 (6.45)	0.841
Mechanical ventilation, *n* (%)	89 ( 57.8)	88 ( 54.7)	0.655	124 ( 66.3)	122 ( 70.5)	0.457
SOFA score, *	5.72 (2.35)	5.79 (2.79)	0.811	6.39 (3.03)	6.38 (2.79)	0.962

**TABLE 3:** Rate of patients with ARDS and the baseline settings of mechanical ventilation in the BMI group.

	Non-overweight (BMI <24)		*p*-value	Overweight (BMI ≥24)		*p*-value
	Placebo group	XBJ group		Placebo group	XBJ group	
N	209	216		132	118	
ARDS, n(%)	66 (31.6)	63 (29.2)	0.663	39 (29.5)	39 (33.1)	0.645
Mechanical ventilation, *n* (%)	129 (61.7)	130 (60.2)	0.822	84 (63.6)	80 (67.8)	0.577
Invasive ventilation, *n* (%)	92 (71.3)	98 (75.4)	0.549	62 (73.8)	54 (67.5)	0.474
Tidal volume, mean (SD)	459.54 (71.36)	462.93 (79.79)	0.719	475.70 (61.10)	484.56 (90.56)	0.462
Positive end-expiratory pressure, mean (SD)	6.43 (2.59)	6.45 (2.80)	0.953	6.10 (2.25)	6.41 (2.74)	0.418
AC, n (%)	4 ( 3.1)	11 ( 8.5)		7 ( 8.3)	3 ( 3.8)	
Bi-level positive airway pressure ventilation, *n* (%)	18 (14.0)	23 ( 17.7)		10 (11.9)	11 (13.8)	
Continuous positive airway pressure, *n* (%)	6 ( 4.7)	4 ( 3.1)		3 ( 3.6)	3 ( 3.8)	
Noninvasive ventilation, *n* (%)	20 (15.5)	17 (13.1)		12 (14.3)	13 (16.2)	
PC	8 ( 6.2)	13 (10.0)		8 ( 9.5)	12 (15.0)	
PSV	9 ( 7.0)	7 ( 5.4)		9 (10.7)	7 ( 8.8)	
SIMV	48 (37.2)	38 (29.2)		26 (31.0)	24 (30.0)	
SIMV+SIMV/AS	0 ( 0.0)	0 ( 0.0)		6 ( 7.1)	4 ( 5.0)	
SIMV+PS	13 (10.1)	15 (11.5)		1 ( 1.2)	0 ( 0.0)	
SPMV+PS	3 ( 2.3)	2 ( 1.5)		2 ( 2.4)	3 ( 3.8)	

ARDS, acute respiratory distress syndrome.

**TABLE 4:** Rate of patients with ARDS and the baseline settings of mechanical ventilation in the FBG group

	Non-hyperglycemia (FBG <7)		*p*-value	Hyperglycemia (FBG ≥7)		*p*-value
	Placebo group	XBJ group		Placebo group	XBJ group	
n	154	161		187	173	
ARDS, n (%)	35 (22.7)	39 (24.2)	0.857	70 ( 37.4)	63 ( 36.4)	0.928
Mechanical ventilation, *n* (%)	89 ( 57.8)	88 ( 54.7)	0.655	124 ( 66.3)	122 ( 70.5)	0.457
Invasive ventilation, *n* (%)	54 ( 60.7)	59 ( 67.0)	0.468	100 ( 80.6)	93 ( 76.2)	0.492
Tidal volume, mean (SD)	468.51 (55.56)	460.09 (89.50)	0.453	464.06 (75.56)	479.16 (80.12)	0.129
Positive end-expiratory pressure, mean (SD)	5.96 (1.93)	6.07 (2.15)	0.713	6.54 (2.76)	6.70 (3.13)	0.678
AC, n (%)	3 ( 3.4)	5 ( 5.7)		8 ( 6.5)	9 ( 7.4)	
Bi-level positive airway pressure ventilation, *n* (%)	13 ( 14.6)	12 ( 13.6)		15 ( 12.1)	22 ( 18.0)	
Continuous positive airway pressure, *n* (%)	8 ( 9.0)	4 ( 4.5)		1 ( 0.8)	3 ( 2.5)	
Noninvasive ventilation, *n* (%)	16 ( 18.0)	18 ( 20.5)		16 ( 12.9)	12 ( 9.8)	
PC	7 ( 7.9)	10 ( 11.4)		9 ( 7.3)	15 ( 12.3)	
PSV	8 ( 9.0)	10 ( 11.4)		10 ( 8.1)	4 ( 3.3)	
SIMV	28 ( 31.5)	25 ( 28.4)		46 ( 37.1)	37 ( 30.3)	
SIMV+SIMV/AS	4 ( 4.5)	3 ( 3.4)		15 ( 12.1)	16 ( 13.1)	
SIMV+PS	1 ( 0.8)	0 ( 0.0)		1 ( 0.8)	0 ( 0.0)	
SPMV+PS	2 ( 2.2)	1 ( 1.1)		3 ( 2.4)	4 ( 3.3)	

In non-overweight and non-hyperglycemia groups, the XueBiJing injection group presented a significant lower 28-day mortality rate (15.3% or 11.2%) than that of the placebo treatment group (24.9% or 20.8%) (*p* = 0.0018 or *p* = 0.03). As for the secondary outcomes, both the duration of mechanical ventilation and total duration of ICU stay presented no statistical difference in non-overweight or non-hyperglycemia patients between XueBiJing injection and placebo treatment groups (Tables 5, 6).

**TABLE 5:** Primary and three secondary outcomes in the BMI group.

BMI group	Non-overweight (BMI <24)		*p*-value	Overweight (BMI ≥24)		*p*-value
	Placebo group	XBJ group		Placebo group	XBJ group	
N	209	216		132	118	
Primary outcome						
Total duration of ICU stay (median [IQR])	11.00 [7.75, 18.00]	11.00 [7.00, 16.25]	0.346	11.00 [8.00, 18.00]	10.00 [7.00, 14.75]	0.103
Secondary outcomes						
The time of mechanical ventilation (median [IQR])	8.00 [5.25, 18.00]	8.00 [6.00, 19.00]	0.806	9.00 [7.00, 17.00]	7.00 [4.00, 12.50]	0.062
28-d mortality, n (%)	52 (24.9)	33 ( 15.3)	0.01863	32 (24.2)	20 ( 16.9)	0.2068

**TABLE 6:** Primary and three secondary outcomes in the FBG group.

FBG group	Non-hyperglycemia (FBG <7)		*p*-value	Hyperglycemia (FBG ≥7)		*p*-value
	Placebo group	XBJ group		Placebo group	XBJ group	
N	154	161		187	173	
Primary outcome						
Total duration of ICU stay (median [IQR])	10.00 [7.00, 16.00]	10.50 [7.00, 14.00]	0.244	11.50 [8.00, 21.75]	10.50 [7.00, 17.00]	0.246
Secondary outcomes						
The time of mechanical ventilation (median [IQR])	8.00 [6.75, 14.25]	8.00 [5.00, 13.00]	0.273	9.00 [6.00, 18.00]	8.00 [5.00, 18.25]	0.69
28-d mortality, n (%)	32 (20.8)	18 ( 11.2)	0.03	52 (27.8)	35 ( 20.2)	0.12

**FIGURE 2:**
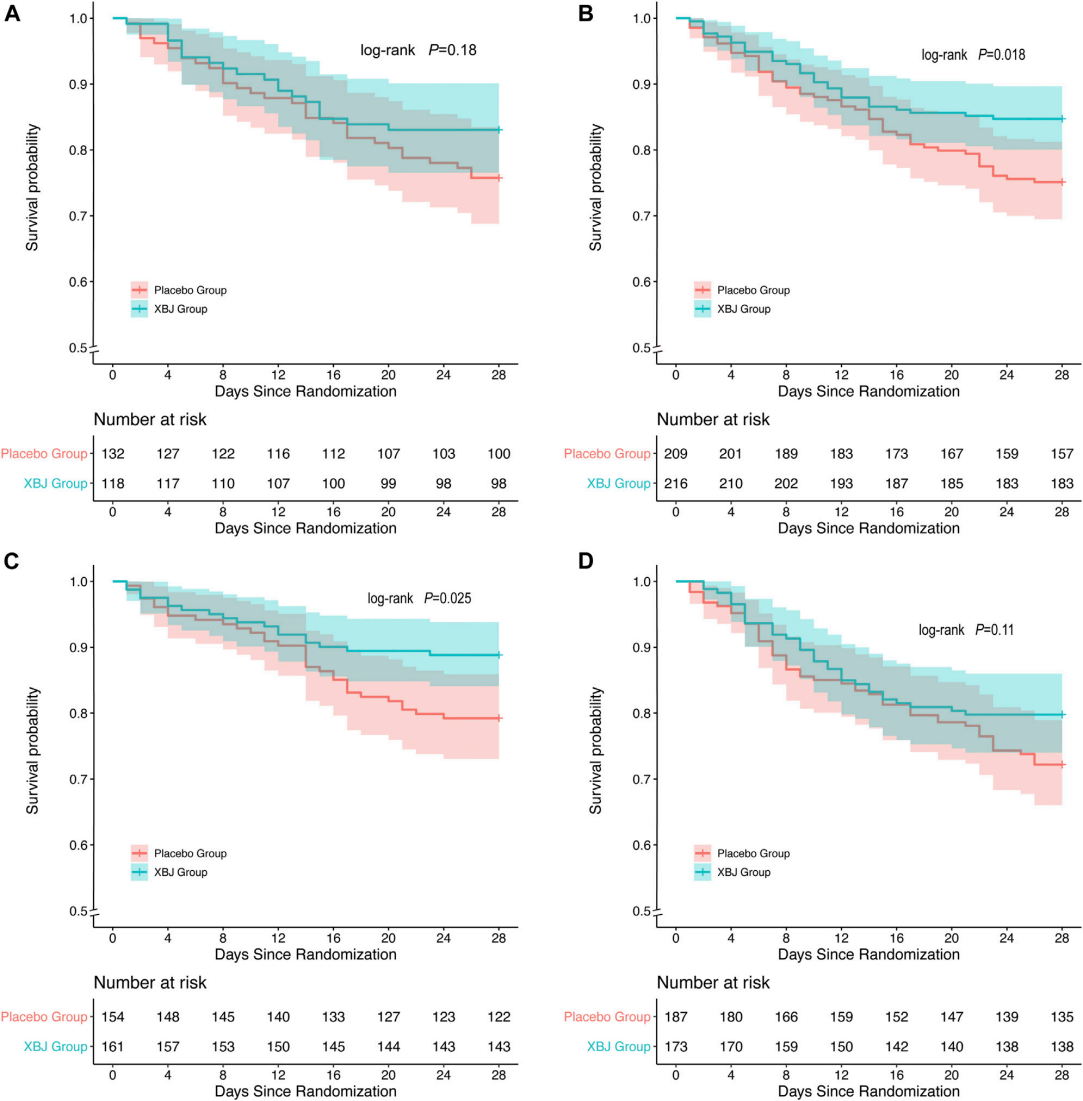
Kaplan–Meier survival curve of overall survival after XueBiJing (XBJ) and placebo for patients in **(A,C)** Patients with XBJ had a significantly inferior overall survival to those who with placebo: non-overweight group: (*p* = 0.018),non-hyperglycemia group (*p* = 0.025) **(B,D)** overweight group: *p* = 0.18, hyperglycemia group: *p* = 0.11

In the non-overweight group (*p* = 0.018) and non-hyperglycemia group (*p* = 0.025), patients with XueBiJing injection had a significantly superior overall survival to those who were treated with placebo. In overweight patients (*p* = 0.18) and hyperglycemia patients (*p* = 0.11), no significant differences in mortality between XueBiJing injection and placebo treatment groups were seen (Figure 2). Some secondary outcomes are listed in Supplementary Tables S1, S2. Among non-overweight or non-hyperglycemia patients, the XueBiJing injection group presented a significantly lower PSI (94.84 ± 28.54 or 86.71 ± 26.81) than the placebo treatment group (102.11 ± 30.18 or 93.89 ± 25.78, *p* = 0.022 or *p* = 0.032) at day 4.

Adverse events (AEs) and clinically significant laboratory abnormalities are summarized in Supplementary Tables S3, S4. There were no significant differences in AEs among patients in the XueBiJing and placebo groups.

## Discussion

The results of the large multicenter trial showed that XueBiJing injection could improve the PSI of patients with SCAP and reduce the 28-day mortality rate compared with those of the placebo group. The 28-day mortality was reduced from 24.63% to 15.87% in patients treated with XueBiJing (*p* < 0.01), which confirmed the effectiveness of XueBiJing injection in the treatment of SCAP.

Our results showed that XueBiJing injection was significantly effective and could reduce the 28-day mortality rate for patients with BMI <24 kg/m^2^ or FBG <7 mmol/L. Even though the 28-day mortality rate for patients with BMI ≥24 kg/m^2^ or FBG ≥7.0 mmol/L presented a lower trend in the XueBiJing injection group, it was not significant. These results suggested that overweight as well as hyperglycemia might have an adverse impact on the efficacy of XueBiJing injection in the treatment of SCAP.

XueBiJing, an injectable prescription from traditional Chinese medicine (TCM), is extensively used in the conventional management of sepsis with a history of more than 10 years (Li et al., 2020). Previous studies have shown that XueBiJing could protect endothelial cells, improve microcirculation and coagulopathy, alleviate inflammatory reaction, and regulate anti-oxidative stress (Xu et al., 2015; Wang et al., 2016; Chen et al., 2017; Zuo et al., 2019). The protective effect appears to be mediated through downregulation of the TLR4 and NF-κB expressions (He et al., 2018). Previous gas chromatography/mass spectrometer (GC/MS)-based metabolomics study demonstrated that XueBiJing could increase the survival rate of the septic animal model by changing serum metabolites, which involved energy metabolism, glucose metabolism, and amino acid metabolism (Jiang et al., 2019).

Obesity is related to the occurrence and development of many diseases. Studies have shown that obesity is related to metabolic dysfunction (Hotamisligil, 2006; Shoelson et al., 2006). Excess adiposity and adipocyte dysfunction can lead to adverse outcomes by altering immune responses through releasing proinflammatory adipokines (Ouchi et al., 2011). Obesity affects lung function and high BMI reduces lung capacity, which are related to the adverse consequences of pneumonia (Rubinstein et al., 1990; Jones and Nzekwu, 2006). Consistent with the reports, our data also showed that obesity patients had a metabolic disorder, as indicated by the higher blood glucose level in the patients with BMI ≥24 kg/m^2^ (8.45 ± 3.82 mmol/L) than that in patients with BMI <24 kg/m^2^ (8.15 ± 3.68 mmol/L). On the other hand, it has been proven that overweight might affect the metabolism of the drug, thereby affecting the therapeutic effect (Hanley et al., 2010; Knibbe et al., 2015). Similarly, our data demonstrated that, not like patients with BMI <24 kg/m^2^, patients with BMI ≥24 kg/m^2^ only had the trend of a lower 28-day mortality rate by XueBiJing injection without significance. Hence, we asserted that BMI ≥24 kg/m^2^ might be one important risk factor for reducing the efficacy of XueBiJing. Therefore, a weight-based XueBiJing dosing strategy might be a better choice and need to be tested with future clinical research studies. Interestingly, there are still epidemiologic studies on general population or critical illness that showed beneficial effects of higher body mass index (BMI) on all-cause mortality (Flegal et al., 2013; Kahlon et al., 2013; Singanayagam et al., 2013). This “obesity survival paradox” and the underlying mechanisms at least need to be tested in SCAP.

SCAP is a disease with significant biochemical and metabolic disturbance (Khardori and Castillo, 2012). In our analysis, we also noticed that certain proportion (50.7%) of patients had the problem of a higher glucose serum level without the present history of diabetes. The poorly regulated glucose metabolism in patients is often associated with increased levels of inflammatory markers (Chang and Yang, 2016). Compared with the non-hyperglycemia group, the hyperglycemia group had much intense inflammation (CRP 77.30 ± 100.20 vs. 96.12 ± 83.38 mg/L) (*p* = 0.016). Hyperglycemia influences a variety of host immune functions, such as chemotaxis, phagocytosis, and bactericidal activity of histiocytic cells (Akbar, 2001; Falguera et al., 2005; Eurich et al., 2010). A previous study already showed the mechanisms that glucose-induced alterations in endothelial cell function promote changes in the basement membrane structure and cause endothelial cell permeability; reactive oxygen species (ROS) have been suggested by others to be common mediators for hyperglycemic cell damage (Simon et al., 2000; Morss and Edelman, 2007). For CAP patients, high glucose serum levels lead to an increased length of hospital stay or higher mortality due to infections. Each 1mmol/L increase of glucose serum levels might result in the risk of in-hospital complications that increased to 3% (0.2–6%) (McAlister et al., 2005; Kornum et al., 2007). Our study also suggested that compared with the non-hyperglycemia group, the 28-day mortality rate in the hyperglycemia group is high, and the duration of ICU stay is long. In distribution studies, the increased level of glycosylation of plasma proteins could reduce the binding of a number of drugs in the blood (Gwilt et al., 1991; Mashayekhi-Sardoo et al., 2019). Hyperglycemia may lead to a decrease in the effect of XueBiJing, so dosage adjustment is required. Our results demonstrated a significantly lower 28-day mortality rate for patients with FBG <7 mmol/L, while only a decreased trend was observed for patients with FBG ≥7.0 mmol/L by XueBiJing injection. Our data suggested that hyperglycemia might have an adverse impact on the efficacy of XueBiJing injection in the treatment of SCAP, indicating that SCAP patients with hyperglycemia might needed to be reconsidered the XueBiJing dose or treatment plan. Better blood glucose level management is strongly recommended.

### Limitations

The results of our study come with some limitations. Although our data come from a well-designed RCT, the analyses were planned post hoc, and the results cannot be considered confirmative. Second, the number of patients included in each group was relatively small and not suitable for multivariate or stratified analysis. A larger sample size would be necessary to validate our conclusions. Mortality is the most robust outcome of a noninferiority trial, but adding more outcomes will be more compelling.

## Conclusion

In this *post hoc* analysis of a large randomized controlled trial, for overweight and hyperglycemia SCAP patients, in the non-overweight group (*p* = 0.018) and non-hyperglycemia group (*p* = 0.025), patients with XueBiJing injection had a significantly superior overall survival to those who were treated with placebo, and the addition of XueBiJing treatment only partially reduced the 28-day mortality but not significantly like the effect in non-overweight and non-hyperglycemia patients. Overweight and hyperglycemic SCAP patients therefore should be identified who are in need of the adjusted dose of XueBiJing injection and intensified care to reduce the risk of death.

## Data Availability

The original contributions presented in the study are included in the article/Supplementary Material, further inquiries can be directed to the corresponding author.

## Abbreviations

APCHE, acute physiology and chronic health evaluation; ARDS, acute respiratory distress syndrome; BMI, body mass index; CAP, community-acquired pneumonia; CRP, C-reactive protein; FBG, fasting blood glucose; ICU, intensive care unit; PSI, pneumonia severity index; RCT, randomized controlled trial; SCAP, severe community-acquired pneumonia; ROS, reactive oxygen species; SOFA, Sequential Organ Failure Assessment; TCM, traditional Chinese medicine.
