# Supplementary material for: Weaker Response to XueBiJing Treatment in Severe Community-Acquired Pneumonia Patients With Higher Body Mass Index or Hyperglycemia: A Post Hoc Analysis of a Randomized Controlled Trial
Source: Front Pharmacol. 2022 Jun 3;13:755536. doi: 10.3389/fphar.2022.755536 (PMC9204492; doi:10.3389/fphar.2022.755536)
Supplement: Supplementary file 1 [file DataSheet1.docx]

**Supplementary Material Table S1. Comparison of the secondary outcomes for the intention-to-treat populations in BMI Group**

|  | **Non-overweight (BMI<24)** | |  | **Overweight (BMI≥24)** | |  |
| --- | --- | --- | --- | --- | --- | --- |
|  | **Placebo Group** | **XBJ Group** | **P-value** | **Placebo Group** | **XBJ Group** | **P-value** |
| **n** | 209 | 216 |  | 132 | 118 |  |
| **PSI score, mean(SD)** |  |  |  |  |  |  |
| **Day 4** | 102.11 (30.18) | 94.84 (28.54) | 0.022 | 96.65 (27.47) | 90.05 (28.80) | 0.097 |
| **Day 8** | 96.70 (32.10) | 89.86 (29.04) | 0.046 | 95.79 (28.27) | 84.23 (31.30) | 0.009 |
| **SOFA, mean (SD)** |  |  |  |  |  |  |
| **Day 4** | 5.12 (3.10) | 4.57 (2.96) | 0.07 | 4.82 (2.52) | 4.43 (2.91) | 0.269 |
| **Day 8** | 4.49 (3.07) | 3.78 (3.13) | 0.026 | 4.35 (2.76) | 3.41 (2.78) | 0.013 |
| **APACHE II score, mean (SD)** |  |  |  |  |  |  |
| **Day 4** | 12.06 (6.27) | 10.98 (6.15) | 0.085 | 11.91 (5.82) | 9.33 (5.43) | 0.001 |
| **Day 8** | 11.09 (6.54) | 9.69 (6.67) | 0.038 | 11.17 (5.79) | 8.24 (5.96) | <0.001 |
| **Procalcitonin, mean (SD)** |  |  |  |  |  |  |
| **Day 4** | 5.23 (18.82) | 6.29 (22.05) | 0.646 | 4.37 (16.58) | 4.23 (14.01) | 0.95 |
| **Day 8** | 5.93 (33.20) | 3.09 (8.45) | 0.319 | 2.66 (5.90) | 1.65 (5.25) | 0.226 |
| **C-reactive protein, mean (SD)** |  |  |  |  |  |  |
| **Day 4** | 62.86 (59.25) | 64.30 (67.92) | 0.844 | 68.20 (69.34) | 52.79 (65.84) | 0.118 |
| **Day 8** | 42.62 (55.17) | 41.48 (51.62) | 0.858 | 57.16 (105.33) | 34.09 (51.18) | 0.062 |
| **D-dimer, mean (SD)** |  |  |  |  |  |  |
| **Day 4** | 2197.76 (3648.44) | 3532.04 (5264.37) | 0.012 | 2452.15 (2882.74) | 3381.82 (6895.58) | 0.219 |
| **Day 8** | 2187.29 (4392.63) | 3053.02 (4811.20) | 0.113 | 2751.51 (3403.20) | 2582.28 (3459.23) | 0.739 |

**Supplementary Material Table S2. Comparison of the secondary outcomes for the intention-to-treat populations in FBG Group**

|  | **non-hyperglycemia(FBG<7)** | |  | **hyperglycemia(FBG≥7)** | |  |
| --- | --- | --- | --- | --- | --- | --- |
|  | **Placebo Group** | **XBJ Group** | **P-value** | **Placebo Group** | **XBJ Group** | **P-value** |
| **n** | 154 | 161 |  | 187 | 173 |  |
| **PSI score, mean(SD)** | |  |  |  |  |  |
| **Day 4** | 93.89 (25.78) | 86.71 (26.81) | 0.032 | 104.86 (30.95) | 99.02 (29.14) | 0.097 |
| **Day 8** | 87.27 (25.65) | 81.73 (26.19) | 0.107 | 103.17 (32.30) | 93.44 (31.96) | 0.012 |
| **SOFA, mean (SD)** |  |  |  |  |  |  |
| **Day 4** | 4.72 (2.54) | 4.07 (2.61) | 0.034 | 5.25 (3.13) | 4.93 (3.17) | 0.356 |
| **Day 8** | 4.07 (2.48) | 3.22 (2.59) | 0.005 | 4.74 (3.26) | 4.07 (3.31) | 0.066 |
| **APACHE II score, mean (SD)** | |  |  |  |  |  |
| **Day 4** | 11.43 (5.97) | 9.69 (5.14) | 0.008 | 12.48 (6.16) | 11.06 (6.56) | 0.041 |
| **Day 8** | 10.18 (5.94) | 8.03 (5.41) | 0.002 | 11.89 (6.41) | 10.28 (7.16) | 0.033 |
| **Procalcitonin, mean (SD)** | |  |  |  |  |  |
| **Day 4** | 4.27 (15.81) | 4.59 (22.55) | 0.898 | 5.44 (19.65) | 6.33 (16.53) | 0.685 |
| **Day 8** | 2.01 (4.62) | 1.55 (4.81) | 0.463 | 7.03 (35.82) | 3.39 (8.99) | 0.267 |
| **C-reactive protein, mean (SD)** | |  |  |  |  |  |
| **Day 4** | 47.19 (51.37) | 52.31 (60.94) | 0.491 | 78.91 (68.22) | 67.57 (72.47) | 0.194 |
| **Day 8** | 44.15 (93.85) | 33.11 (44.14) | 0.264 | 51.77 (61.39) | 43.67 (57.27) | 0.292 |
| **D-dimer, mean (SD)** |  |  |  |  |  |  |
| **Day 4** | 1938.99 (4123.83) | 2535.71 (3937.47) | 0.267 | 2610.83 (2493.33) | 4324.14 (7169.62) | 0.01 |
| **Day 8** | 2063.35 (4887.05) | 2589.29 (4090.02) | 0.387 | 2727.54 (3051.82) | 3135.50 (4580.34) | 0.407 |

**Supplementary Material TABLE S3. Adverse events and Clinically Significant Laboratory Abnormalities in BMI Group**

| **Characteristics** | **Non-overweight (BMI<24)** | | |  | **Overweight (BMI≥24)** | |  |  |
| --- | --- | --- | --- | --- | --- | --- | --- | --- |
|  | **Placebo Group** | **n(%)** | **XBJ Group** | **n(%)** | **Placebo Group** | **n(%)** | **XBJ Group** | **n(%)** |
| **Clinically Significant Laboratory Abnormalities** | 82 | 29.5 | 101 | 36.3 | 53 | 19.1 | 42 | 15.1 |
| **Low red blood cell count** | 10 | 3.6 | 16 | 5.8 | 8 | 2.9 | 7 | 2.5 |
| **High red blood cell count** | 1 | 0.4 | 2 | 0.7 | 1 | 0.4 | 0 | 0 |
| **Low hemoglobin count** | 10 | 3.6 | 5 | 1.8 | 4 | 1.4 | 3 | 1.1 |
| **High hemoglobin count** | 1 | 0.4 | 0 | 0 | 0 | 0 | 0 | 0 |
| **Low white blood cell count** | 2 | 0.7 | 4 | 1.4 | 0 | 0 | 1 | 0.4 |
| **High white blood cell count** | 14 | 5 | 13 | 4.7 | 5 | 1.8 | 6 | 2.2 |
| **Low platelet count** | 6 | 2.2 | 4 | 1.4 | 3 | 1.1 | 5 | 1.8 |
| **High platelet count** | 5 | 1.8 | 4 | 1.4 | 2 | 0.7 | 1 | 0.4 |
| **Low hematocrit levels** | 4 | 1.4 | 3 | 1.1 | 1 | 0.4 | 2 | 0.7 |
| **High hematocrit levels** | 10 | 3.6 | 8 | 2.9 | 4 | 1.4 | 1 | 0.4 |
| **Elevated alanine aminotransferase** | 13 | 4.7 | 12 | 4.3 | 14 | 5 | 10 | 3.6 |
| **Decreased aspartate amino transferase** | 2 | 0.7 | 1 | 0.4 | 0 | 0 | 0 | 0 |
| **Elevated aspartate amino transferase** | 11 | 4 | 12 | 4.3 | 8 | 2.9 | 8 | 2.9 |
| **Decreased total bilirubin** | 0 | 0 | 2 | 0.7 | 0 | 0 | 0 | 0 |
| **Elevated total bilirubin** | 2 | 0.7 | 10 | 3.6 | 6 | 2.2 | 4 | 1.4 |
| **Decreased blood urea nitrogen** | 1 | 0.4 | 1 | 0.4 | 0 | 0 | 0 | 0 |
| **Elevated blood urea nitrogen** | 6 | 2.2 | 6 | 2.2 | 6 | 2.2 | 2 | 0.7 |
| **Decreased serum creatinine** | 5 | 1.8 | 7 | 2.5 | 2 | 0.7 | 0 | 0 |
| **Elevated serum creatinine** | 12 | 4.3 | 8 | 2.9 | 5 | 1.8 | 5 | 1.8 |
| **Elevated blood glucose** | 4 | 1.4 | 12 | 4.3 | 7 | 2.5 | 4 | 1.4 |
| **Decreased K+** | 7 | 2.5 | 4 | 1.4 | 1 | 0.4 | 2 | 0.7 |
| **Elevated K+** | 4 | 1.4 | 0 | 0 | 1 | 0.4 | 0 | 0 |
| **Decreased Na+** | 1 | 0.4 | 0 | 0 | 1 | 0.4 | 1 | 0.4 |
| **Elevated Na+** | 5 | 1.8 | 3 | 1.1 | 1 | 0.4 | 2 | 0.7 |
| **Decreased plasma fibrinogen** | 3 | 1.1 | 0 | 0 | 0 | 0 | 0 | 0 |
| **Elevated plasma fibrinogen** | 3 | 1.1 | 8 | 2.9 | 3 | 1.1 | 3 | 1.1 |
| **Decreased prothrombin time** | 1 | 0.4 | 1 | 0.4 | 0 | 0 | 0 | 0 |
| **Elevated prothrombin time** | 5 | 1.8 | 5 | 1.8 | 4 | 1.4 | 0 | 0 |
| **Decreased activated partial thromboplastin time** | 0 | 0 | 1 | 0.4 | 0 | 0 | 0 | 0 |
| **Elevated activated partial thromboplastin time** | 4 | 1.4 | 3 | 1.1 | 1 | 0.4 | 3 | 1.1 |
| **Elevated fibrin D-dimer** | 2 | 0.7 | 4 | 1.4 | 4 | 1.4 | 0 | 0 |
| **Elevated C reactive protein** | 0 | 0 | 0 | 0 | 0 | 0 | 1 | 0.4 |
| **Elevated procalcitonin** | 0 | 0 | 0 | 0 | 1 | 0.4 | 0 | 0 |

**Supplementary Material TABLE S4. Adverse events and Clinically Significant Laboratory Abnormalities in FBG Group**

| **Characteristics** | **non-hyperglycemia(FBG<7)** | | |  | **hyperglycemia(FBG≥7)** | |  |  |
| --- | --- | --- | --- | --- | --- | --- | --- | --- |
|  | **Placebo Group** | **n(%)** | **XBJ Group** | **n(%)** | **Placebo Group** | **n(%)** | **XBJ Group** | **n(%)** |
| **Clinically Significant Laboratory Abnormalities** | 55 | 19.8 | 55 | 19.8 | 80 | 28.8 | 88 | 31.7 |
| **Low red blood cell count** | 11 | 4 | 13 | 4.7 | 7 | 2.5 | 10 | 3.6 |
| **High red blood cell count** | 2 | 0.7 | 1 | 0.4 | 0 | 0 | 1 | 0.4 |
| **Low hemoglobin count** | 6 | 2.2 | 5 | 1.8 | 8 | 2.9 | 3 | 1.1 |
| **High hemoglobin count** | 0 | 0 | 0 | 0 | 1 | 0.4 | 0 | 0 |
| **Low white blood cell count** | 0 | 0 | 3 | 1.1 | 2 | 0.7 | 2 | 0.7 |
| **High white blood cell count** | 4 | 1.4 | 4 | 1.4 | 15 | 5.4 | 15 | 5.4 |
| **Low platelet count** | 3 | 1.1 | 2 | 0.7 | 6 | 2.2 | 7 | 2.5 |
| **High platelet count** | 2 | 0.7 | 2 | 0.7 | 5 | 1.8 | 3 | 1.1 |
| **Low hematocrit levels** | 2 | 0.7 | 3 | 1.1 | 3 | 1.1 | 2 | 0.7 |
| **High hematocrit levels** | 6 | 2.2 | 4 | 1.4 | 8 | 2.9 | 5 | 1.8 |
| **Elevated alanine aminotransferase** | 10 | 3.6 | 9 | 3.2 | 17 | 6.1 | 13 | 4.7 |
| **Decreased aspartate amino transferase** | 2 | 0.7 | 1 | 0.4 | 0 | 0 | 0 | 0 |
| **Elevated aspartate amino transferase** | 8 | 2.9 | 6 | 2.2 | 11 | 4 | 14 | 5 |
| **Decreased total bilirubin** | 0 | 0 | 0 | 0 | 0 | 0 | 2 | 0.7 |
| **Elevated total bilirubin** | 1 | 0.4 | 7 | 2.5 | 7 | 2.5 | 7 | 2.5 |
| **Decreased blood urea nitrogen** | 1 | 0.4 | 1 | 0.4 | 0 | 0 | 0 | 0 |
| **Elevated blood urea nitrogen** | 4 | 1.4 | 4 | 1.4 | 8 | 2.9 | 4 | 1.4 |
| **Decreased serum creatinine** | 4 | 1.4 | 4 | 1.4 | 3 | 1.1 | 3 | 1.1 |
| **Elevated serum creatinine** | 10 | 3.6 | 4 | 1.4 | 7 | 2.5 | 9 | 3.2 |
| **Elevated blood glucose** | 0 | 0 | 0 | 0 | 11 | 4 | 16 | 5.8 |
| **Decreased K+** | 2 | 0.7 | 4 | 1.4 | 3 | 1.1 | 5 | 1.8 |
| **Elevated K+** | 3 | 1.1 | 0 | 0 | 2 | 0.7 | 0 | 0 |
| **Decreased Na+** | 1 | 0.4 | 1 | 0.4 | 1 | 0.4 | 0 | 0 |
| **Elevated Na+** | 3 | 1.1 | 2 | 0.7 | 3 | 1.1 | 3 | 1.1 |
| **Decreased plasma fibrinogen** | 1 | 0.4 | 0 | 0 | 2 | 0.7 | 0 | 0 |
| **Elevated plasma fibrinogen** | 3 | 1.1 | 6 | 2.2 | 3 | 1.1 | 5 | 1.8 |
| **Decreased prothrombin time** | 0 | 0 | 1 | 0.4 | 1 | 0.4 | 0 | 0 |
| **Elevated prothrombin time** | 3 | 1.1 | 3 | 1.1 | 6 | 2.2 | 2 | 0.7 |
| **Decreased activated partial thromboplastin time** | 0 | 0 | 0 | 0 | 0 | 0 | 1 | 0.4 |
| **Elevated activated partial thromboplastin time** | 1 | 0.4 | 1 | 0.4 | 4 | 1.4 | 5 | 1.8 |
| **Elevated fibrin D-dimer** | 3 | 1.1 | 3 | 1.1 | 3 | 1.1 | 1 | 0.4 |
| **Elevated C reactive protein** | 0 | 0 | 1 | 0.4 | 0 | 0 | 0 | 0 |
| **Elevated procalcitonin** | 1 | 0.4 | 0 | 0 | 0 | 0 | 0 | 0 |

**Supplementary Material TABLE S4.The chemical composition of XueBiJing**

| **Compound** | **Molecular Mass** | **Molecular Formula** | **Average content level** |
| --- | --- | --- | --- |
| **Constituents originating from the component herbs Chuanxiong/Danggui only** | **Da** |  | **μmol/L** |
| **Senkyunolide I** | **224.1049** | **C12H16O4** | **293.1±28.1** |
| **Senkyunolide H** | **224.1049** | **C12H16O4** | **64.8±6.3** |
| **Senkyunolide G** | **208.1099** | **C12H16O3** | **44.5±3.1** |
| **Senkyunolide N** | **226.1205** | **C12H18O4** | **40.8±5.2** |
| **3-Hydroxy-3-n-butylphthalide** | **206.0943** | **C12H14O3** | **37.6±4.8** |
| **Z-6,7-Epoxyligustilide** | **206.0943** | **C12H14O3** | **12.7±0.5** |
| **6,7-Dihydroxyligustilide** | **224.1049** | **C12H16O4** | **10.9±1.1** |
| **Senkyunolide A** | **192.1150** | **C12H16O2** | **4.8±2.6** |
| **Senkyunolide J** | **226.1205** | **C12H18O4** | **3.4±0.4** |
| **4-Hydroxy-3-n-butylphthalide**  **Constituents originating from the component herb Chishao only** | **206.0943** | **C12H14O3** | **2.4±0.3** |
| **Mudanpioside F** | **344.1471** | **C16H24O8** | **5.8±1.0** |
| **1-O-β-D-Glucopyranosyl-Paeonisuffrone** | **360.1420.** | **C16H24O9** | **1.6±0.5** |
| **Desbenzoylpaeoniflorin** | **376.1369** | **C16H24O10** | **22.1±13.7** |
| **Albiflorin** | **480.1632** | **C23H28O11** | **102.4±27.6** |
| **Paeoniflorin** | **480.1632** | **C23H28O11** | **2470±142.8** |
| **Oxypaeoniflorin** | **496.1581** | **C23H28O12** | **112.9±7.6** |
| **Oxypaeoniflorin isomer** | **496.1581** | **C23H28O12** | **4.0±0.8** |
| **Ortho-oxypaeoniflorin** | **496.1581** | **C23H28O12** | **4.3±0.7** |
| **Mudanpioside E** | **526.1686** | **C24H30O13** | **11.3±1.1** |
| **6’-O-Galloyl-desbenzoylpaeoniflorin** | **528.1479** | **C23H28O14** | **1.3±0.5** |
| **Benzoylpaeoniflorin** | **584.1894** | **C30H32O12** | **58.3±6.8** |
